# Correlation Between LPS and LTA Levels With Clinical Features in Secondary Endodontic Infections

**DOI:** 10.1111/aej.12963

**Published:** 2025-07-02

**Authors:** Ederaldo P. Godoi, Pedro Ivo G. Fagundes, Priscila A. Francisco, Maraísa G. Delboni, Marlos Barbosa‐Ribeiro, Marcos S. Endo, Erica M. Lopes, Frederico C. Martinho, Brenda P. F. A. Gomes

**Affiliations:** ^1^ Department of Restorative Dentistry, Endodontics Division, Piracicaba Dental School State University of Campinas‐UNICAMP Piracicaba SP Brazil; ^2^ School of Dentistry Universidade de Pernambuco ‐ Arcoverde Pernambuco PR Brazil; ^3^ Department of Dentistry, Health Sciences Center Uningá – Ingá University Center Maringá PR Brazil; ^4^ Division of Endodontics, Department of Advanced Oral Sciences and Therapeutics, School of Dentistry University of Maryland Baltimore USA

**Keywords:** endodontics, endotoxin, lipoteichoic acid periapical disease, LPS, LTA, periapical disease, retreatment, virulence factors

## Abstract

This study aimed to quantify LPS and LTA levels in root canals requiring endodontic retreatment due to secondary or persistent infection and evaluate the relationship between Gram‐positive and Gram‐negative virulence factors and clinical symptoms and signs. Samples were collected from the root canals of 40 patients who needed endodontic retreatment and had radiographic evidence of apical periodontitis. Lipopolysaccharides (LPS) and lipoteichoic acid (LTA) were quantified using Limulus amebocyte lysate and enzyme‐linked immunosorbent assay tests, respectively. LPS and LTA were detected in all samples, with mean values being 5.1 EU/mL(±2.83) and 537.53 pg/mL(±134.14), respectively. Significant correlations were found between levels of LPS and LTA with clinical features. In conclusion, coronal restoration directly influences the load of LPS and LTA in root canals with secondary infection. Furthermore, higher concentrations of endotoxin (LPS) are associated with larger areas of periapical bone resorption, as well as with the presence of clinical signs and symptoms.

## 1 Introduction

Decontamination of the root canal system plays a crucial role in the success of endodontic therapy since the presence of remaining microorganisms and their byproducts is the main etiological factor of endodontic failure [1, 2]. Studies indicate that the persistence of virulence factors after endodontic therapy may have a negative impact on endodontic prognosis, triggering and perpetuating periapical inflammation [3, 4].

Specific virulence factors are associated with the pathogenicity of microorganisms detected in primary and secondary/persistent endodontic infections [4, 5, 6].

Endotoxins (lipopolysaccharide/LPS) present in the outer membrane of Gram‐negative microorganisms associated with endodontic failure, such as *Porphyromonas gingivalis, Fusobacterium nucleatum, Prevotella nigrescens*, and *Treponema denticola* , can contribute to the course of endodontic infection, as they act on the host's immune system, stimulating the release of pro‐inflammatory cytokines already associated with the development of painful symptoms and the induction of bone resorption [3, 7, 8]. Moreover, the LPS structure shows considerable heterogeneity among different bacterial species, thus activating host cells in different ways [9].

Lipoteichoic acid (LTA), present in the cell wall of Gram‐positive microorganisms frequently detected in cases of secondary/persistent infection, such as *Enterococcus faecalis, Parvimonas micra, Filifactor alocis*, and *Gemella morbillorum* [10, 11, 12, 13] is responsible for developing an important role in the adhesion of bacteria to dental surfaces, favouring bacterial colonisation and biofilm formation. In addition, it acts on the host's immune system through the stimulation of macrophages and consequent release of cytokines and metalloproteinases, presenting some similar properties to LPS from Gram‐negative bacteria [4, 11, 14].

Previous studies have evaluated the response of the host immune system in the presence of bacterial species, as well as in the presence of isolated LPS molecules, reporting the development of a similar inflammatory reaction in both conditions [14, 15, 16]. This fact highlights the importance of understanding not only the bacteriological profile of endodontic infections but also the profile of Gram‐positive and Gram‐negative virulence factors and their relationship with the course of secondary endodontic infections.

Despite advances in the understanding of endodontic infections, the relationship between clinical features and the presence of virulence factors in root canals with secondary infection remains poorly explored. Therefore, this study has two main objectives: to quantify LPS and LTA levels in root canals requiring endodontic retreatment due to secondary or persistent infection and to assess the relationship between Gram‐positive and Gram‐negative virulence factors and the manifestation of clinical signs and symptoms. Additionally, it aims to evaluate the influence of clinical factors, such as the quality of restoration and obturation, on the load of these virulence factors.

## 2 Material and Methods

### 2.1 Patient Selection

This work was approved by the Research Ethics Committee of the Piracicaba Dental School (FOP/UNICAMP) under protocol #018/2014 and according to regulatory resolution #466/2012 of the National Health Council. The Human Research Ethics Committee of the Piracicaba Dental School, State University of Campinas – UNICAMP, Piracicaba, SP, Brazil, approved this work (CAAE 86140218.0.0000.5418), describing the sampling methods. Patients signed an informed consent form prior to their participation in the study.

**FIGURE 1:**
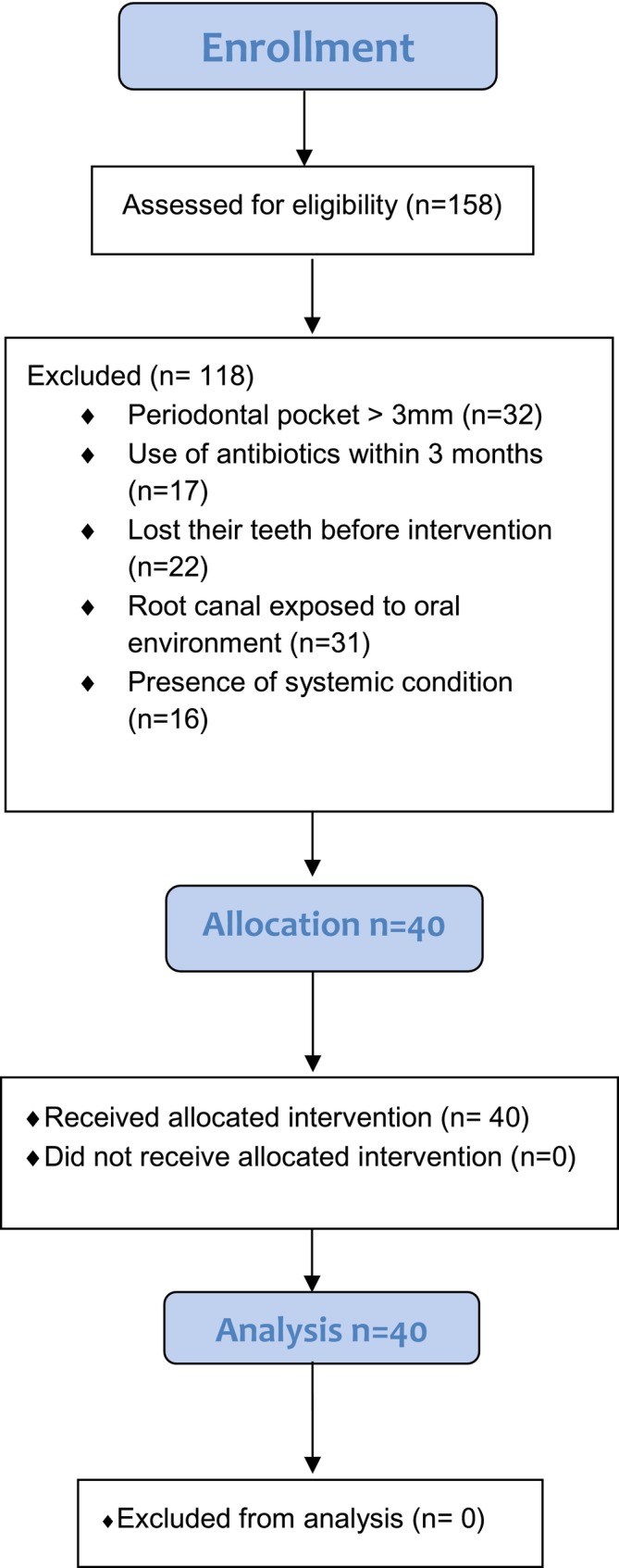
Flow diagram representing the selection of patients for the study.

A cross‐sectional study was conducted following the Strengthening of the Reporting of Observational Studies in Epidemiology (STROBE) statement. Initially, all clinical records and radiographs of patients referred for retreatment at the Piracicaba Dental School were investigated. Figure 1 shows a flow diagram comprising the volunteers assessed for eligibility. Forty patients were selected.

The participants' ages ranged from 19 to 69 years old. All teeth (*n* = 40) had been previously treated endodontically and showed radiographic evidence of apical periodontitis, which should be understood as the loss of continuity of the lamina dura in the periapical region of the tooth or as the presence of a radiolucent region adjacent to the apex of the affected tooth.

The area of the periradicular radiolucency present on the initial radiographic examination was recorded using the mean values of three consecutive measurements using ImageJ software (National Institutes of Health, Bethesda, MD) as previously described [17].

Failure of root canal treatment was determined by two calibrated observers based on radiographic and clinical findings. Of the 40 teeth included, all had been previously root‐filled and showed radiographic evidence of apical periodontitis associated with defective root canal filling (e.g., radiolucent spaces in the filling due to poorly compacted gutta‐percha; root canal filling limit shorter than or more than 2 mm from the radiographic apex), defective coronal restoration (e.g., if there were open margins, fracture, or recurrent decay), and persistent symptoms (e.g., spontaneous pain; pain on palpation or tenderness to percussion; and persistent sinus tract).

Exclusion criteria were as follows: patients who received antibiotic therapy in the past 3 months, who had severe systemic disease (American Society of Anesthesiologists [ASA]‐III), who had teeth that could not be properly isolated with a rubber dam, who had an absence of coronal restoration, and who had periodontal pockets ≥ 3 mm.

### 2.2 Clinical Procedures and Sample Collections

The methodology was used according to previous investigations [8, 10, 11, 18]. A dental operating microscope (DF Vasconcelos S.A., São Paulo, SP, Brazil) was used during all clinical procedures and sample collection.

All the materials (e.g., high‐speed burs, endodontic files, absorbent paper points, dental instruments) used in the study were sterilised by gamma radiation with cobalt 60 (20 KGy for 6 h) to eliminate preexisting endotoxins. The teeth were isolated with a rubber dam. The crown and surrounding structures were disinfected with 30% hydrogen peroxide (vol/vol for 30 s) followed by 2.5% sodium hypochlorite (NaOCl) for the same period and then inactivated with 5% sodium thiosulphate. The disinfection of these sites was monitored by taking a swab sample from both the external and internal surfaces of the crown and the surrounding areas. The swab was moistened in 50 μL of saline solution to facilitate spreading before being streaked onto a plate containing 5% defibrinated sheep blood and fastidious anaerobe agar (FAA, LAB M; Heywood, Lancashire, UK), which were incubated anaerobically and aerobically for up to 14 days. Next, DNA was extracted from the swab, and polymerase chain reaction (PCR) analysis was performed using universal bacterial primers. If any positive culture or DNA amplification product was obtained, the case was excluded from the study.

The sampling procedures were performed according to previous studies [11, 12, 18]. A two‐stage access preparation was performed under anaesthesia with 2% lidocaine and epinephrine at a concentration of 1:100000. The access cavity was made without the use of water spray but under manual irrigation with LPS‐free water (LAL water, Lonza) and by using a sterile high‐speed diamond bur, with the first stage intended to promote a major removal of contaminants. In the second stage, before entering the pulp chamber, the access cavity was disinfected and checked for contaminants as previously described. All procedures were performed aseptically. Root filling materials were removed from the working length based on preoperative radiographs. Reciproc R25 files (VDW, Munich, Germany) were used in a crown‐down technique according to the manufacturer's instructions and with no chemical solution. In the case of multi‐rooted teeth, the samples were collected from the largest root canal associated with a radiographic image of apical periodontitis to concentrate the investigation on microbiological/virulence factors related to a single ecological environment. Additionally, in these cases, Coltosol (Vigodent, Coltene, Rio de Janeiro, RJ, Brazil) was placed at the canal entrances during sample collection to avoid cross‐contamination.

Before collecting samples from the root canal, a #20 K‐file (Dentsply‐Maillefer, Ballaigues, Switzerland) was used to determine the working length with the assistance of an apex locator (Novapex, Forum Technologies, Rishon le‐Zion, Israel), which was set to zero. Two sterile/pyrogen‐free paper points (Dentsply‐Maillefer) were consecutively inserted into the full length of the root canal and left in position for 60 s. All the paper points were removed and placed into an empty pyrogen‐free glass tube, which was stored at −80°C until LPS/LTA quantification.

The status of the root canal (i.e., dry canal or presence of exudate) was checked during sample collection. In the case of a dry canal, LPS‐free water (LAL water, Lonza) was used to moisten the root canals to allow better sample collection. In the case of a wet canal (or those that have been previously irrigated with LPS‐free water) many paper points as needed were used to absorb all the fluid inside the canal. The teeth were excluded from the study whenever the paper point could not reach the full length of the root canal.

### 2.3 Quantification of LPS Levels

The methodology was used according to previous investigations [12, 18], in which LPS quantification was performed by using the Pyrogent‐5000 turbidimetric limulus amebocyte lysate (LAL) test (Lonza, Walkersville, MD). This test uses an amebocyte lysate preparation in combination with a photometric incubator and appropriate software for the photometric detection of endotoxins. As a parameter for the calculation of LPS in the root canal samples, a standard curve was established with *Escherichia coli* endotoxins supplied by the kit at a known concentration (100 EU/1 mL^−1^) and then prepared in dilutions (0.01 EU/mL; 0.10 EU/mL; 1 EU/mL; 10 EU/mL; 100 EU/mL) according to the manufacturer's instructions.

All reactions were performed in duplicate for validation of the test. A 96‐well microplate (Corning Costas, Cambridge, MA) was kept in a 37°C heating block throughout the assay. LPS samples were first suspended in 1 mL of LAL water, shaken in a vortex mixer for 1 min, and serially diluted to 10 μL. Immediately after, 100 μL of blank was added to a 96‐well microplate at concentrations of 0.01, 0.10, 1, and 10 EU mL‐ 1 according to the standard endotoxin solution with 100 μL of samples. The procedure was performed following the manufacturer's instructions. Endotoxin absorbance in the samples was measured individually in each well using an enzyme‐linked immunosorbent assay (ELISA) microplate reader (Bio‐Rad Laboratories Inc., Hercules, CA) at 340 nm. The WinKQCL software (Lonza) was used to automatically provide a linear log/log correlation of the reaction time of each standard with the corresponding endotoxin concentration of each sample.

### 2.4 Quantification of LTA Levels

The methodology was used according to previous studies [11, 12], in which the LTA levels in the samples were measured by using a human LTA ELISA kit (My BioSource, San Diego, CA). All reagents used in the process were supplied in the kit. Briefly, the sample, standard, and control were added to a well plate, precoated with LTA‐specific monoclonal antibody, and incubated at 37°C for 90 min. The plate was washed twice with buffer solution, and anti‐LTA antibody labelled with biotin was added to the well and incubated at 37°C for 60 min. After washing three times, the enzyme working solution was added to the wells, and the plate was incubated at 37°C for 30 min. Next, the plate was washed five times, the colour reagent solution was added to the wells, and the plate was incubated at 37°C for 30 min in a dark environment. The stop solution was then added, and within 10 min, the LTA levels were evaluated by an ELISA plate reader with an optical density set at 450 nm. The assay was normalised for negative control values. Each optical density value, expressed as mean and standard deviation, was obtained from two independent experiments done in triplicate.

### 2.5 Statistical Analysis

Initially, all clinical and radiographic data collected during treatment sessions were subjected to a descriptive analysis of frequency and percentage (e.g., age, gender, number of roots, time elapsed since primary treatment, presence of symptoms stimulated by vertical percussion or apical palpation, previous pain, quality of obturation, quality of restoration and lesion size). For descriptive purposes, the central tendency of quantitative data was expressed and analysed through mean, standard deviation, minimum value, and maximum value. The size of the radiographic periapical lesion was categorised as > 4 mm or < 4 mm, based on the average size of periapical lesions found in the present study.

Given the normality of the values obtained in the quantification of endotoxin (LPS) and lipoteichoic acid (LTA) using the Shapiro–Wilk statistical test (*p* > 0.05), differences in the mean concentration of LPS and LTA were evaluated among various clinical conditions (e.g., age, gender, number of roots, presence of symptoms stimulated by vertical percussion or apical palpation, previous pain, size of the radiographic periapical lesion, obturation quality, and restoration quality). These differences were assessed using the independent t‐test.

## 3 Results

### 3.1 Microbial Assessment

The disinfection protocol of the operatory field and the teeth's surrounding structures was proven to be effective as no microbial growth on the control plates after 14 days and no DNA amplification products were evident on the agarose gels.

### 3.2 Clinical and Radiographic Features

This study included 40 teeth referred for endodontic retreatment due to chronic apical periodontitis following prior treatment. Among the teeth analysed, 36/40 (90%) were single‐rooted, 3/40 (7.5%) were multi‐rooted, and 1/40 (2.5%) had two roots. Regarding location in the mouth, 28/40 (70%) were found in the upper arch, while 12/40 (30%) were in the lower arch.

Most participants in this study were female (28/40%–70%), while 30% (12/40) were male. The ages of the patients ranged from 19 to 69 years, with an average age of approximately 48 years. 47.5% (19/40) of the participants were aged 48 years or younger, and 52.5% (21/40) were older than 48 years. The average time since the initial endodontic treatment was around 12 years, with a minimum reported time of 2 years and a maximum of 35 years.

As for symptoms, 12.5% (5/40) of the patients reported a history of previous pain, while 50% (20/40) responded positively to vertical percussion testing. None of the volunteers experienced tenderness to palpation (0/40). There were no cases of fistulas or facial oedema. The periodontal probing depth in all surfaces of the teeth analysed ranged from 1 to 3 mm in 100% of the cases (40/40).

The size of the periapical lesions varied from 2 to 14 mm, with an average size of 4.75 mm. Among these, 50% (20/40) had lesions smaller than 4 mm, while the remaining 50% (20/40) had lesions equal to or greater than 4 mm in size. Root canal filling quality was rated as satisfactory in 27.5% (11/40) of cases, while 72.5% (29/40) exhibited deficiencies in the length and/or quality of root canal filling.

Coronal sealing quality was considered satisfactory in 30% (12/40) cases. However, it was unsatisfactory in 70% (28/40) of cases, which exhibited clinical and/or radiographic signs of coronal microleakage and poor adaptation of restorative materials.

Statistical analysis revealed no significant correlations between patient gender, age, dental group, or the anatomical location of the evaluated teeth and the clinical or radiographic characteristics, nor with the LTA or LPS level observed. Detailed frequency and percentage distribution of the clinical and radiographic data analysed in this study can be found in Table 1.

**TABLE 1:** Frequency and percentage distribution of clinical and radiographic features.

Variable	Category	Frequency and Percentage
Gender	Male	12 (30%)
	Female	28 (70%)
Age	≤ 48 years	19 (47.5%)
	> 48 years	21 (52.5%)
Number of roots	Single‐rooted	36 (90%)
	Bi‐rooted	1 (2.5%)
	Multi‐rooted	3 (7.5%)
Tooth localisation	Upper	28 (70%)
	Lower	12 (30%)
Dental Group	Central incisor	13 (32.5%)
	Lateral incisor	12 (30%)
	Canine	3 (7.5%)
	Premolar	9 (2.5%)
	Molar	3 (7.5%)
Periodontal probing	≤ 3 mm	40 (100%)
	> 3 mm	0 (0%)
Spontaneous pain	Present	0 (0%)
	Absent	40 (100%)
Previous pain	Present	5 (12.5%)
	Absent	35 (87.5%)
Sinus tract	Present	0 (0%)
	Absent	40 (100%)
Tenderness to percussion	Present	20 (50%)
	Absent	20 (50%)
Pain on palpation	Present	0 (0%)
	Absent	40 (100%)

Swelling	Present	0 (0%)
	Absent	40 (100%)
Exudate	Present	8 (20%)
	Absent	32 (80%)
Periapical lesion	Present	40 (100%)
	Absent	0 (0%)
Lesion size	< 4 mm	20 (50%)
	≥ 4 mm	20 (50%)
Root filling quality	Good	11 (27.5%)
	Poor	29 (72.5%)
Coronal restoration quality	Good	12 (30%)
	Poor	28 (70%)

#### 3.2.1 Quantification of Lipopolysaccharide/Endotoxin (LPS) Levels in Root Canals

The exploratory analysis confirmed the normality of the obtained data (Shapiro–Wilk). Endotoxins were detected in 100% of the samples analysed using the LAL assay, with a mean concentration of 5.1 EU/mL (±2.83).

#### 3.2.2 Quantification of Lipoteichoic Acid (LTA) Levels in Root Canals

The exploratory analysis confirmed the normality of the obtained data (Shapiro–Wilk). Lipoteichoic acid (LTA) molecules were detected in 100% of the analysed samples using the ELISA immunoenzymatic assay, with a mean concentration of 537.53 pg/mL (±134.14).

#### 3.2.3 Correlation of LPS and LTA Levels With Signs and Symptoms

The impact of clinical factors on LPS levels in the samples was confirmed. The quality of coronal sealing significantly influenced the LPS levels detected in the root canals (*p* < 0.05).

The presence of exudate after root canal filling removal, tenderness to percussion, and the radiographic periapical lesion size were associated with higher levels of LPS (*p* < 0.05). Table 2 shows the positive associations between clinical features and LPS levels.

**TABLE 2:** Positive associations between clinical features and LPS levels.

		LPS (EU/mL)	p‐value
Coronal sealing quality	Good	3.7 (±0.72)A	0,0378
	Poor	5.7 (±0.525)B	
Tenderness to percussion	Present	6.32 (±0.49) A	0,0047
	Absent	3.87 (±0.65) B	
Exudate	Present	7.17 (±0.56)A	0,0186
	Absent	4.57 (±0.5)B	
Lesion size	≤ 4 mm	3.0 (±0.38)A	< 0,0001
	> 4 mm	7.18 (±0.46)B	

*Note:* Different uppercase letters indicate the presence of a statistical difference between the means obtained in the study groups (*p* < 0.05).

Clinical features and LTA levels were correlated (*p* < 0.05). Coronal sealing is shown to be a critical factor influencing LTA concentration in cases of endodontic failure (*p* < 0.05). No significant correlation was found between LTA levels and the development of signs and symptoms of secondary/persistent infection. The correlation between LTA levels and coronal sealing quality can be found in Table 3.

**TABLE 3:** Mean and standard deviation of LTA levels and the coronal sealing quality.

	Coronal sealing quality	
	Good	Poor
LTA (pg/mL)	319.5 (±54.09) A	586.0 (±20.37) B

*Note:* Different uppercase letters indicate the presence of a statistical difference between the means obtained in the study groups (*p* < 0.05).

## 4 Discussion

The failure of primary endodontic treatment is characterised by the persistence or development of chronic apical periodontitis [8, 11, 18], with microbial factors, such as the presence of microorganisms and their byproducts, described as the main etiological factors of endodontic failure [1, 2].

In the present study, 80% of the cases exhibited varied and often overlapping clinical features, including previous pain, tenderness to percussion, and exudate, which guided the indication for retreatment in line with established endodontic criteria. For the remaining cases, retreatment was based on persistent periapical lesions observed radiographically beyond two years post‐treatment, in accordance with literature suggesting that such lesions are unlikely to resolve spontaneously. Importantly, in several of these asymptomatic cases, retreatment was also motivated by planned prosthetic rehabilitation. In these instances, eliminating potential residual infection prior to post and crown placement was considered essential to ensure long‐term success and stability of the prosthetic treatment [13, 19].

The variation in time elapsed between initial treatment and retreatment (2–35 years) in the present work reflects real‐world clinical practice, where retreatment is sought at different times after primary treatment. To reduce bias, we used strict clinical and radiographic criteria, focusing on current pathology rather than time since treatment. Although two years may seem short for an outcome assessment, literature supports its adequacy. Periapical healing typically begins within the first year following treatment, with significant radiographic changes often becoming evident by the second year. In cases of persistent infection, studies have shown that lesion size may decrease within 18 months after retreatment, regardless of whether a single‐visit or multiple‐visit protocol was used [20]. The ESE S3‐level clinical practice guideline [21] also recommends reassessment within at least one year.

The microbiological profile associated with cases of endodontic failure has been extensively investigated over the years, characterised through classical microbiology methods [1, 11, 22] and molecular biology [10, 13, 18, 23]. Advances in microbial identification methods have revealed the predominance of a mixed and heterogeneous community composed of Gram‐positive and Gram‐negative strict anaerobic and facultative microorganisms in cases of persistent secondary infection [8, 10, 13, 18, 23]. However, few studies address the influence of microbial byproducts in the course of persistent secondary infections [5, 11, 12].

Studies reported that even after the death of bacterial cells, the persistence of residual bacterial byproducts in the root canal can induce a chronic inflammatory response, leading to endodontic failure [4, 11, 24] in cases of inadequately treated and/or restored teeth.

The primary objective of coronal sealing is to establish a barrier against salivary microleakage, preventing the recontamination of the root canal. Numerous studies emphasise its influence on endodontic prognosis, considering it a determining factor for the success of endodontic therapy [25].

The epidemiological analysis of clinical data found in this study reaffirms the importance of coronal sealing, as out of the 40 cases with secondary/persistent infection included, 28 cases (70%) had deficient restorations. Additionally, a significant increase in LPS and LTA levels in the analysed canals was observed when associated with inadequate restorations. This finding is consistent with previous studies that linked the presence of inadequate restorations to a greater diversity of microbial species [8], highlighting the influence of clinical factors on the microbial community in teeth with secondary endodontic infections.

The ability of LPS and LTA to indirectly cause harm to the host organism is documented in the literature [4, 26]. The interaction between bacterial byproducts and the host organism modulates the secretion of inflammatory biomarkers directly associated with the course and maintenance of periapical inflammation [27].

Higher levels of LPS have been correlated with cases displaying more pronounced signs of infection, such as tenderness to percussion, exudate in the root canal, and larger areas of periapical bone resorption, consistent with other studies [5, 18, 28].

The correlation observed between tenderness to percussion and elevated levels of LPS can be linked to an increase in PGE‐2 secretion promoted by the host organism [26]. This biomarker is associated with vasodilation, increased vascular permeability, and collagen degradation [29]. Higher levels of LPS and PGE‐2 were found in the supernatants of macrophages stimulated with bacterial content from teeth with primary infections that exhibited tenderness to percussion and/or palpation, symptoms capable of signalling the presence of periapical inflammation [28].

The increase in vascular permeability indirectly promoted by LPS can also explain the correlation detected between the presence of exudate in the root canal during sample collection and elevated LPS levels. Exudate is defined as a fluid composed of cells and plasma proteins that escapes from the vascular system and accumulates in tissues, typically as a result of inflammation. The presence of exudate within the root canal is indicative of an active inflammatory process in the periapical region [30].

Studies conducted through cell culture describe the ability of LPS to stimulate bone resorption [31, 32]. Clinical research also supports the findings of this study, correlating higher levels of endotoxin with larger areas of periapical bone resorption [6, 7, 33]. LPS released from infected root canals triggers the synthesis of IL‐1 and TNF‐alpha, which, in turn, stimulates the production of Matrix Metalloproteinase (MMP) by macrophages to promote periapical bone resorption. Furthermore, LPS stimulates bone resorption by increasing RANKL (receptor activator of NF‐κB ligand) through Toll‐like receptor‐2 activation in osteoblasts, inhibiting osteoblast differentiation.

This study highlights the critical role of rehabilitative treatment in the overall success of endodontic therapy. The presence of bacterial components such as lipoteichoic acid (LTA) and lipopolysaccharide (LPS) in root canals suggests their involvement in modulating the periradicular inflammatory response, which may contribute to the persistence of apical lesions and the manifestation of clinical symptoms [34]. These findings emphasise the need for meticulous clinical and restorative protocols to reduce the risk of residual infection and promote long‐term treatment success [34].

Moreover, the host immune response is a key determinant of treatment outcomes. The interaction between microbial products like LTA and LPS and the host's immune system shapes the periapical inflammatory environment. While an effective immune response fosters resolution and healing, an imbalanced or exaggerated reaction can lead to chronic inflammation and delayed recovery [27, 28, 33, 34]. A deeper understanding of these host–pathogen interactions is therefore essential for the development of therapeutic approaches that not only control infection but also modulate the immune response to optimise healing and enhance the prognosis of endodontic therapy.

## 5 Conclusion

In conclusion, the quality of coronal restoration significantly influences the levels of LPS and LTA in root canals with secondary infection. Both LPS and LTA were detected in high concentrations in cases indicated for endodontic retreatment, reflecting the persistent microbial challenge in these cases. Higher concentrations of endotoxins, particularly LPS, were strongly associated with larger areas of periapical bone resorption, the presence of clinical signs and symptoms, including percussion pain and the presence of exudate. These findings underscore the importance of proper coronal sealing and effective infection control to minimise the risk of periapical inflammation and the manifestation of clinical symptoms.

## Author Contributions

Ederaldo P. Godoi Jr and Brenda P. F. A. Gomes: conceptualisation. Ederaldo P. Godoi Jr, Priscila A. Francisco, Marlos Barbosa Ribeiro, Maraísa G. Delboni, Marcos S. Endo, Erica M. Lopes and Brenda P. F. A. Gomes: methodology. Ederaldo P. Godoi Jr, Pedro Ivo G. Fagundes, Erica M. Lopes and Brenda P. F. A. Gomes: validation. Ederaldo P. Godoi Jr, Erica M. Lopes and Brenda P. F. A. Gomes: formal analysis. Ederaldo P. Godoi Jr, Pedro Ivo G. Fagundes, Priscila A. Francisco, Marlos Barbosa Ribeiro, Maraísa G. Delboni, Marcos S. Endo, Erica M. Lopes and Brenda P. F. A. Gomes: investigation. Ederaldo P. Godoi Jr and Brenda P. F. A. Gomes: resources. Ederaldo P. Godoi Jr, Fagundes Pedro Ivo G., Erica M. Lopes and Brenda P. F. A. Gomes: data curation. Ederaldo P. Godoi Jr, Erica M. Lopes and Brenda P. F. A. Gomes: writing – original draft. Ederaldo P. Godoi Jr, Erica M. Lopes and Brenda P. F. A. Gomes: writing – review and editing preparation. Ederaldo P. Godoi Jr, Erica M. Lopes, Frederico C. Martinho and Brenda P. F. A. Gomes: visualisation. Brenda P. F. A. Gomes: supervision. Brenda P. F. A. Gomes: project administration. Brenda P. F. A. Gomes: funding acquisition.

## Disclosure

We affirm that we have no financial affiliation (e.g., employment, direct payment, stock holdings, retainers, consultantships, patent licensing arrangements or honoraria), or involvement with any commercial organisation with direct financial interest in the subject or materials discussed in this manuscript, nor have any such arrangements existed in the past three years. Any other potential conflict of interest is disclosed. The informed consent of all human subjects who participated in the experimental investigation reported or described in this manuscript was obtained after the nature of the procedure and possible discomforts and risks had been fully explained.

## Data Availability

The data that support the findings of this study are available from the corresponding author upon reasonable request.
